# Supplementary material for: MetaQTL: a package of new computational methods for the meta-analysis of QTL mapping experiments
Source: BMC Bioinformatics. 2007 Feb 8;8:49. doi: 10.1186/1471-2105-8-49 (PMC1808479; doi:10.1186/1471-2105-8-49)
Supplement: Additional File 7 — MetaQTL Package : jar file and tutorial. This Zip archive contains both the MetaQTL JAR file and the files of the tutorial. [file 1471-2105-8-49-S7.zip › org.inra.metaqtl/doc/org/metaqtl/util/GeneticMapProperties.html]

GeneticMapProperties


|  |  |  |
| --- | --- | --- |
| **Overview** \| **Package** \| **Class** \| **Use** \| **Tree** \| **Deprecated** \| **Index** \| **Help** | |  |
| PREV CLASS   **NEXT CLASS** | **FRAMES**    **NO FRAMES**     **All Classes** |  |
| SUMMARY: NESTED \| FIELD \| CONSTR \| METHOD | DETAIL: FIELD \| CONSTR \| METHOD |  |


---


## org.metaqtl.util Class GeneticMapProperties

```
java.lang.Object
  org.metaqtl.util.GeneticMapProperties
```

---

``` public final class GeneticMapProperties extends java.lang.Object ```

General comments must be added here.

**Author:**
:   Julien Cornouiller
    Additions must be added here.

---

**Constructor Summary**

`GeneticMapProperties()`


| **Method Summary** | |
| --- | --- |
| `static CrossPopulation` | `getCrossDesign(IBioGenome map)`             This routine returns an object which handle the properties of the offspring which have been used to create the genetic map. |
| `static CrossPopulation` | `getMappingCross(IBioGenome map)` |
| `static int` | `getMappingFunction(IBioGenome map)`             This methods returns the integer code of the mapping function associated to the given genetic map. |
| `static int` | `getMappingUnit(IBioGenome map)` |
| `static boolean` | `needToBeRescaled(IBioGenome map)`             Returns true if the given map need to be rescaled - due to the mapping extansion for some pedigree designs. |
| `static void` | `setCrossDesign(IBioGenome map, CrossPopulation crossDesign)` |
| `static void` | `setMappingContext(IBioGenome map, int mapping_function, int mapping_unit)` |

**Methods inherited from class java.lang.Object**

`clone, equals, finalize, getClass, hashCode, notify, notifyAll, toString, wait, wait, wait`

**Constructor Detail**

### GeneticMapProperties

```
public GeneticMapProperties()
```


**Method Detail**

### getMappingFunction

```
public static int getMappingFunction(IBioGenome map)
```

:   This methods returns the integer code of the mapping
    function associated to the given genetic map.

    :   **Returns:**: the mapping function integer code. **See Also:**: `IConsensusConstants, IGeneticMapProperties.`

---


### getCrossDesign

```
public static CrossPopulation getCrossDesign(IBioGenome map)
```

:   This routine returns an object which handle the
    properties of the offspring which have been used
    to create the genetic map. This object is created
    from the cross properties of the genetic map.

    :   **Parameters:**: `map` - a genetic map **Returns:**: the cross population type for this genetic map **See Also:**: `IConsensusConstants, IGeneticMapProperties.`

---


### getMappingUnit

```
public static int getMappingUnit(IBioGenome map)
```

:   **Parameters:**: `map` - **Returns:**

---


### needToBeRescaled

```
public static boolean needToBeRescaled(IBioGenome map)
```

:   Returns true if the given map need to be rescaled - due
    to the mapping extansion for some pedigree designs.

    :   **Parameters:**: `map` - **Returns:**

---


### setMappingContext

```
public static void setMappingContext(IBioGenome map,
                                     int mapping_function,
                                     int mapping_unit)
```

:   **Parameters:**: `map` -: `mapping_function_haldane` -: `mapping_unit_cm` -

---


### setCrossDesign

```
public static void setCrossDesign(IBioGenome map,
                                  CrossPopulation crossDesign)
```

:   **Parameters:**: `map` -: `crossDesign` -

---


### getMappingCross

```
public static CrossPopulation getMappingCross(IBioGenome map)
```

:   **Parameters:**: `map` - **Returns:**


---


|  |  |  |
| --- | --- | --- |
| **Overview** \| **Package** \| **Class** \| **Use** \| **Tree** \| **Deprecated** \| **Index** \| **Help** | |  |
| PREV CLASS   **NEXT CLASS** | **FRAMES**    **NO FRAMES**     **All Classes** |  |
| SUMMARY: NESTED \| FIELD \| CONSTR \| METHOD | DETAIL: FIELD \| CONSTR \| METHOD |  |


---
